# Critical behavior of density-driven and shear-driven reversible–irreversible transitions in cyclically sheared vortices

**DOI:** 10.1038/s41598-021-98959-w

**Published:** 2021-09-29

**Authors:** S. Maegochi, K. Ienaga, S. Okuma

**Affiliations:** Department of Physics, Tokyo Institute of Technology, 2-12-1 Ohokayama, Meguro-ku, Tokyo, 152-8551 Japan

**Keywords:** Physics, Condensed-matter physics, Phase transitions and critical phenomena

## Abstract

Random assemblies of particles subjected to cyclic shear undergo a reversible–irreversible transition (RIT) with increasing a shear amplitude *d* or particle density *n*, while the latter type of RIT has not been verified experimentally. Here, we measure the time-dependent velocity of cyclically sheared vortices and observe the critical behavior of RIT driven by vortex density *B* as well as *d*. At the critical point of each RIT, $B_{\mathrm {c}}$ and $d_{\mathrm {c}}$, the relaxation time $\tau$ to reach the steady state shows a power-law divergence. The critical exponent for *B*-driven RIT is in agreement with that for *d*-driven RIT and both types of RIT fall into the same universality class as the absorbing transition in the two-dimensional directed-percolation universality class. As *d* is decreased to the average intervortex spacing in the reversible regime, $\tau (d)$ shows a significant drop, indicating a transition or crossover from a loop-reversible state with vortex-vortex collisions to a collisionless point-reversible state. In either regime, $\tau (d)$ exhibits a power-law divergence at the same $d_{\mathrm {c}}$ with nearly the same exponent.

## Introduction

Nonequilibrium phase transitions have been studied intensively in these decades^1–3^. A typical example is a reversible–irreversible transition (RIT), which is observed in many-particle systems under cyclic shear^4–10^. When random assemblies of particles are cyclically sheared with a shear amplitude *d*, collisions between the particles cause the system to self-organize into a relatively ordered configuration where less collisions occur. This phenomenon is called the random organization^5,7,11–14^. For small *d*, the system reaches a reversible steady state where all particles return to their initial position after each shear cycle, whereas above a threshold value $d_{\mathrm {c}}$, the particles finally settle into an irreversible state where the particle motion is diffusive and the reversibility is lost. At $d_{\mathrm {c}}$, the relaxation time $\tau$ to reach the steady state diverges as a power law^5,7,10^ whose exponent $\nu$ is very close to the value expected for a directed percolation (DP) universality class of an absorbing phase transition^1,2^.

**Figure 1:**
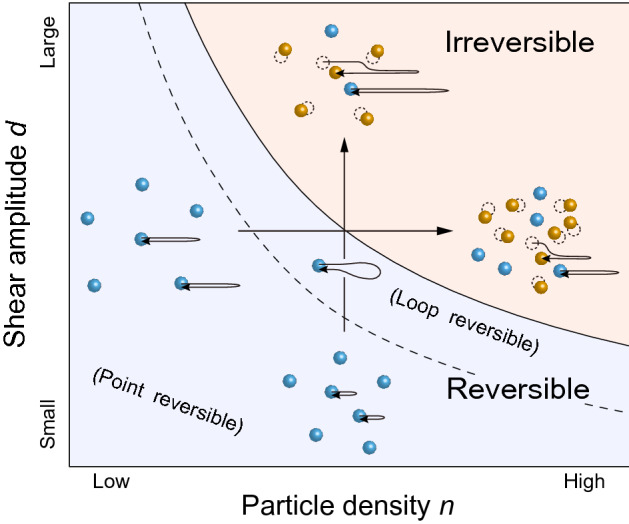
Schematic phase diagram of RIT in the *n* and *d* plane. The solid line marks RIT. The reversible state is located in the low-*n* and small-*d* region, while the irreversible state lies in the high-*n* and large-*d* region. The upward and rightward arrows represent the shear-driven and density-driven RIT, respectively. The dashed line shows the boundary between the collisionless point-reversible state and the loop-reversible state with particle collisions. Inset: The dotted and closed circles, respectively, indicate the particle positions at the beginning and end of a driving cycle. Blue and orange circles denote the reversible and irreversible particles, respectively. Representative particle trajectories are schematically illustrated by arrows starting from particles.

Figure 1 shows a schematic phase diagram of RIT drawn based on colloidal experiments^4,15^ and simulations^16–19^, in which the phase boundary of RIT represented by the solid line is crossed vertically as shown by an upward arrow. This diagram indicates that RIT also occurs by increasing a particle density *n* at fixed *d* as shown by a rightward arrow. This raises an interesting question of whether the RIT driven by *n* (density-driven RIT) is in the same universality class as the RIT driven by *d* (shear-driven RIT). Within the linear approximation, the critical behavior characterized by the critical exponents would be independent of the driving parameter. In fact, numerical studies employing different computational models have reported similar values of critical exponents both for the shear-driven RIT^5,20^ and the density-driven RIT^21,22^.

We consider, however, that this is not trivial in actual systems. Some experiments studying critical phenomena of various phase transitions^23,24^ have reported that the critical exponents of the transition depend on the control parameters that drive the transition. To our knowledge, the critical behavior of the density-driven RIT has not yet been studied experimentally. This is probably because in systems such as colloidal suspensions^5,25^ and emulsions^26,27^ where RIT has been intensively studied, it is difficult to conduct measurements in which the particle density *n* is changed in a controllable manner.

In this work, we study the critical behavior of the density-driven RIT in a superconducting vortex system, where the vortices behave as two-dimensional (2D) monodisperse many particles with repulsive interaction. We use an amorphous (*a*-)$\hbox {Mo}_{{x}}\hbox {Ge}_{1-x}$ film with weak random pinning that gives rise to random local shear. The density of vortices $n (\propto B)$ can be controlled precisely by an applied magnetic field *B*. In the same vortex system, we have recently observed the critical behavior of the shear-driven RIT^10^, which allows a direct comparison of the critical behaviors between the two types of RIT. We measure the transient voltage *V*(*t*) induced by cyclically sheared vortex motion in various *B* at fixed *d* and observe a monotonic increase in the amplitude |*V*(*t*)| and a relaxation toward a steady-state voltage $V^{\infty }$, indicative of the random organization. The relaxation time $\tau (B)$ to reach the steady state exhibits a power-law divergence at a threshold field $B_{\mathrm {c}}$, indicating the density-driven RIT. Two kinds of critical exponents extracted from |*V*(*t*)| are, within error bars, in agreement with the values obtained from the shear-driven RIT and with the theoretical ones expected for the absorbing transition, demonstrating that both types of RIT belong to the same universality class as 2D DP.

We have also found that as *d* is decreased in the reversible regime ($d < d_{\mathrm {c}}$) for the shear-driven RIT^10^, $\tau (d)$ shows a sharp, significant reduction at a shear amplitude $d_{\mathrm {c}1}$ which is close to the average intervortex spacing $a_{0}$. This finding, together with the prediction from recent simulations^16–19^, indicates a transition or crossover from a loop-reversible state with vortex-vortex collisions to a collisionless point-reversible state, as schematically shown by the dashed line in Fig. 1. In either regime, $\tau (d)$ exhibits a power-law divergence at the same critical point of RIT with the same exponent.

## Results

We used a 330-nm thick strip-shaped film of *a*-$\hbox {Mo}_{{x}}\hbox {Ge}_{1-x}$ with weak pinning, which was the same sample as used in our previous work for the shear-driven RIT^10^. Detailed information about the sample is described in “Methods” section. The field *B* was applied perpendicular to the film surface at 4.1 K and the vortices generated by *B* were driven by applying a current. The vortex motion with the average velocity *v* induces the voltage $V = vBl$, which was measured by using voltage probes spaced $l =$ 1.2 mm apart.

For shearing experiments in the strip-shaped superconductor, a random pinning potential due to quenched disorder plays an important role. When the vortices periodically driven by an ac current pass close to the pinned vortices or unoccupied pinning centers, they feel a repulsive or an attractive force, respectively. The magnitude of each force decreases with increasing the distance from pinning centers, thus giving rise to the random local shear around the pinning centers^6,10,28,29^.

In our previous work for the shear-driven RIT^10^, the experiment was conducted at 3.5 T, where the depinning current shows a small peak just prior to melting of the vortex lattice. In the so-called peak-effect regime^30–36^, the effective pinning that the moving vortices feel is so strong that random organization due to local shear around the pinning centers is very pronounced. In this work for the density-driven RIT, *B* was changed from 2.0 to 4.2 T in the peak-effect regime and the corresponding mean intervortex spacing $a_0 \approx \sqrt{\Phi _0/B}$ ranged from 35 to 24 nm, where $\Phi _0$ is the flux quantum. The size of the vortex core is estimated to be $\approx 1 \times 10$ nm.

For the experiment on RIT, a disordered initial vortex configuration is required to realize the random organization. Therefore, we drive the vortices for a long time with $d = 3\,\upmu$m, which is much larger than $d_{\mathrm {c}} \approx 45$ nm^10^. This protocol leads to the randomization of the vortex configuration owing to the diffusivity in the irreversible state. Then, the vortices are subjected to the ac drive of constant $d = 60$ nm: for detail, see “Methods” section. Our experiments using such procedures correspond to the critical-quench experiments in other systems showing the DP criticality^1,2,37,38^.

**Figure 2:**
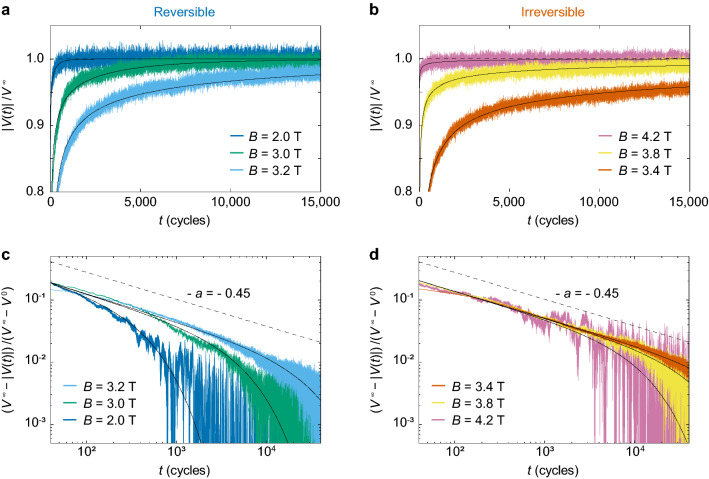
Random organization of cyclically sheared vortices around RIT. (**a**,**b**) The time evolution of the voltage responses $$|V(t)|/V^{\infty }$$ for the disordered initial configuration subjected to the ac drive with the fixed shear amplitude of $$d = 60$$ nm for various fields: (**a**) $$B=$$ 2.0, 3.0, and 3.2 T, and (**b**) $$B=$$ 4.2, 3.8, and 3.4 T from top to bottom. For clarity, vertical lines of the individual ac voltage pulses are removed from the graphs and only the amplitude of the pulse is shown. Horizontal dashed lines mark the steady-state value $$|V(t)|/V^{\infty } = 1$$. (**c**,**d**) Replots of the data shown in (**a**,**b**) as $$(V^{\infty }-|V(t)|)/(V^{\infty }-V^{0})$$ versus *t* on a double logarithmic scale: (**c**) $$B = $$ 3.2, 3.0, and 2.0 T, and (**d**) $$B = $$ 3.4, 3.8, and 4.2 T from top to bottom. Dashed lines in (**c**,**d**) denote the slope of $$-a=-0.45$$ expected for the DP theory in 2D^1,2^. Solid lines in (**a**–**d**) indicate the fits to Eq. (1).

Shown in Fig. 2a,b are the representative voltage responses $|V(t)|/V^{\infty }$ of the system to the ac drive with $d = 60$ nm for different $B =$ 2.0, 3.0, 3.2 T and $B =$ 4.2, 3.8, 3.4 T from top to bottom, respectively, where |*V*(*t*)| is normalized by $V^{\infty }(\equiv |V(t\rightarrow \infty )|)$ in the steady state and *t* corresponds to the number of cycles. For clarity, vertical lines of the individual ac voltage pulses are removed from the graphs and only the amplitude of the pulse, |*V*(*t*)|, is shown. To clearly see the difference between several voltage curves, the large voltage region, $|V(t)|/V^{\infty }>$ 0.8, is enlarged and shown. It is commonly observed that $|V(t)|/V^{\infty }$ increases monotonically toward the steady-state value of unity. This behavior is explained in terms of the random organization: Initially, a vortex flow is a disordered flow, where the vortices cannot move easily due to frequent collisions and hence |*V*(*t*)| is small at $t\sim 0$. With an increase in the number of shear cycles, the collided vortices rearrange into more organized and mobile configurations. Thus, |*V*(*t*)| increases monotonically toward $V^{\infty }$, where the system arrives at a less disordered state in which the vortices are easier to move than in the initial state.

The $|V(t)|/V^{\infty }$ curves in Fig. 2a indicate that the relaxation is longer for higher *B*, while those in Fig. 2b show the longer $\tau$ for lower *B*. This indicates that $\tau (B)$ takes a maximum value around $B =$ 3.2–3.4 T. We extract $\tau$ using the following function presented in^5,39^:

1 $$\begin{aligned} |V(t)| = V^{\infty } - (V^{\infty } - V^{0})\exp {(-t/\tau )/t^{a}}, \end{aligned}$$

where $V^{0}$ and $V^{\infty }$ are the initial and steady-state voltages, respectively, and $\tau$ is the characteristic time at which the relaxation crosses over from a power-law decay with an exponent *a* to an exponential decay, as seen in Fig. 2c,d. Hence, *a* is relevant very close to the transition where $\tau \rightarrow \infty$. We first determine the exponent *a* from Fig. 2c,d, where we replot all the data shown in Fig. 2a,b as $(V^{\infty } - |V(t)|)/(V^{\infty } - V^{0})$ versus *t* on a double logarithmic scale, respectively. The replotted data near RIT, namely for $B =$ 3.2 and 3.4 T, exhibit a power-law decay over a wide range of *t*, as indicated by dashed lines with a slope of $-0.45$. Thus, we obtain the exponent $a = 0.45 \pm 0.05$ independently of another fitting parameter $\tau$. It is known that *a* is the critical exponent of the DP class^1,2^. The theory of DP predicts that the time dependence of the order parameter, i.e., the fraction of active (irreversible) particles, in the case of RIT, obeys the power law with the exponent *a* at the critical point. The value $a = 0.45 \pm 0.05$ obtained here is in agreement with the theoretical value $a \approx 0.45$ for the 2D DP universality class^1,2^. We have also obtained a similar value $a = 0.40 \pm 0.05$ for the shear-driven RIT^10^.

**Figure 3:**
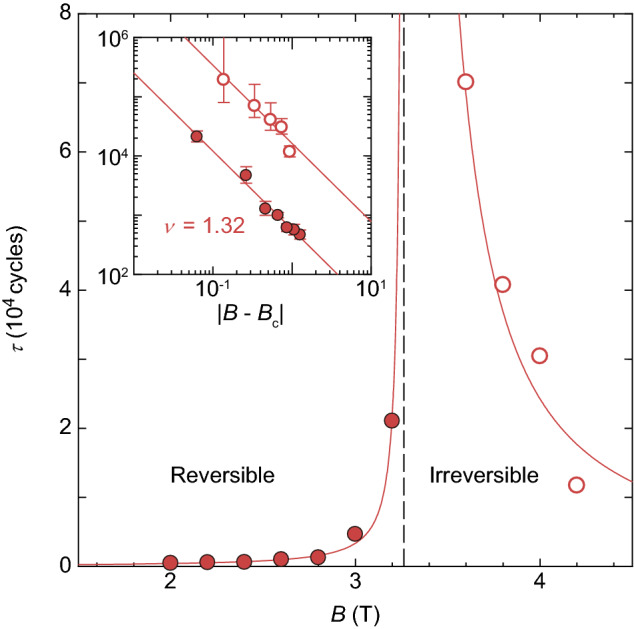
The critical divergence of the relaxation time $\tau (B)$ at the density-driven RIT. The closed and open symbols represent $\tau$ for $B\le$ 3.2 T and $B\ge$ 3.4 T, respectively, showing a power-law divergence at $B_{\mathrm {c}} = 3.26$ T from both sides, as marked by a vertical dashed line, where $B_{\mathrm {c}}$ indicates the density-driven RIT. Inset: $\log {{\tau }}$ versus $\log {{|B-B_{\mathrm {c}}|}}$, where the symbols correspond to those in the main panel. Error bars correspond to the fitting errors resulting from the uncertainty in determining *a*. The red lines both in the main panel and inset are the power-law fits by $\tau \propto |B- B_{\mathrm{c}}|^{-\nu }$ with $\nu = 1.32 \pm 0.10$. The value of $\nu = 1.32 \pm 0.10$ is, within errors, in agreement with the theoretical value of $\nu = 1.295 \pm 0.006$ expected for the DP universality class in 2D^1,2^.

The solid lines in Fig. 2a–d show the results of the fits to Eq. (1) using $a = 0.45$. In Fig. 3, $\tau$ extracted from the fits is plotted as a function of *B*. Closed and open circles represent the data for $B \le$ 3.2 T and $B \ge$ 3.4 T, respectively. We find a critical divergence of $\tau$ at $B_{\mathrm {c}} = 3.26$ T, as marked by a vertical dashed line, indicating the density-driven RIT. A corresponding intervortex spacing at $B_{\mathrm {c}}$ is $a_{\mathrm {0c}} \approx 27$ nm. The inset of Fig. 3 shows the log-log plot of $\tau$ versus $|B-B_{\mathrm {c}}|$ for all the data taken, including the data shown in the main panel, where symbols are the same as in the main panel. The red lines both in the main panel and the inset represent the power-law fits by $\tau \propto |B - B_{\mathrm {c}}|^{-\nu }$, where the best fits are obtained with $\nu = 1.32 \pm 0.10$. This value is again close to the value obtained for the shear-driven RIT, $\nu = 1.38 \pm 0.08$^10^, and the predicted one, $\nu = 1.295 \pm 0.006$, for the 2D DP class^1,2^. These results clearly show that the critical behavior of RIT is independent of the parameters, *B* and *d*, that drive the transition and that RIT falls into the same universality class as the absorbing phase transition^1,2,40,41^ in 2D DP models.

## Discussion

**Figure 4:**
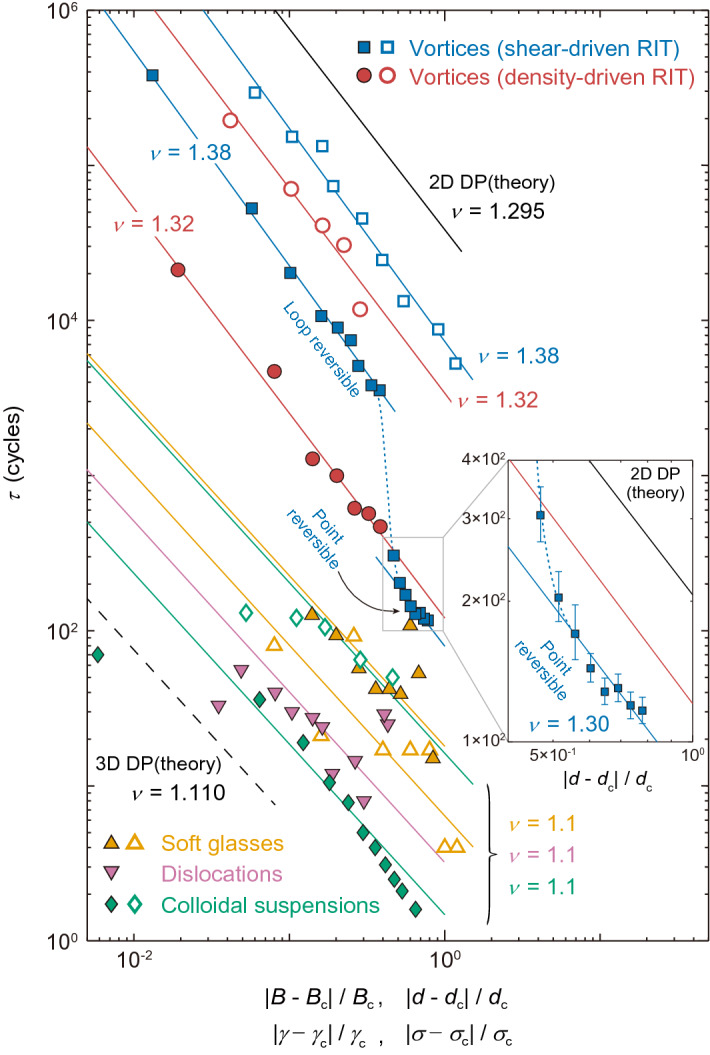
The critical behavior of $\tau$ in different systems. The log-log plots of $\tau$ versus the dimensionless driving parameters near the shear-driven RIT reported in colloidal suspensions (green diamonds)^5^, soft glasses (yellow upright triangles)^42^, and dislocations in small crystals (magenta inverted triangles)^43^, along with our data near the density-driven (red circles) and shear-driven (blue squares) RIT. The quantities, $|B-B_{\mathrm {c}}|/B_{\mathrm {c}}$, $|d-d_{\mathrm {c}}|/d_{\mathrm {c}}$, $|\gamma -\gamma _{\mathrm {c}}|/\gamma _{\mathrm {c}}$, and $|\sigma -\sigma _{\mathrm {c}}|/\sigma _{\mathrm {c}}$, for the horizontal axis represent the normalized distance from the critical point, where $\gamma$ indicates the strain amplitude for colloidal suspensions and soft glasses, and $\sigma$ represents the stress for dislocations. Closed and open symbols denote the data in the reversible and irreversible states, respectively. Colored solid lines represent the power-law fits with critical exponents $\nu$ shown in the figure. We extract the following critical values: $B_{\mathrm {c}} = 3.26$ T and $d_{\mathrm{c}} = 45.2$ nm^10^, $\gamma _{\mathrm{c}} = 1.71$^5^, $\gamma _{\mathrm {c}} = 0.25$^42^, and $\sigma _{\mathrm {c}} = 390$ MPa^43^. Black solid and dashed lines represent slopes of $\nu =$ 1.295 and 1.110, respectively, which are predicted by the DP theory in 2D and 3D^1,2^. The blue dotted line is a guide to the eye. The sharp, significant drop of $\tau$ (closed blue squares) at $d_{\mathrm {c}1}(=$ 25 nm) indicates the transition or the crossover from the loop-reversible to point-reversible state in the reversible phase for the vortex system. Inset: $\log {\tau }$ versus $\log {(|d-d_{\mathrm {c}}|/d_{\mathrm {c}})}$ for the small *d* region in the reversible phase of the shear-driven RIT for the vortex system is enlarged and shown. The symbols and lines are the same as those in the main panel. The solid blue line represents the power-law fit by $\tau \propto |d- d_{\mathrm {c}}|^{-\nu }$ with $\nu = 1.30 \pm 0.18$ in the point-reversible regime $(d < d_{\mathrm {c}1})$. Error bars correspond to the fitting errors resulting from the uncertainty in determining *a*.

To our knowledge, this work is the first experimental demonstration of the density-driven RIT with critical behaviors, while the shear-driven RIT has been reported in different many-particle systems. To compare the critical behaviors of $\tau$ observed in different systems, in Fig. 4, we display the log-log plots of $\tau$ versus the dimensionless driving parameters near the shear-driven RIT reported in colloidal suspensions^5^, soft glasses^42^, and dislocations in small crystals^43^, along with our data near the density-driven and shear-driven RIT. The quantities, $|B-B_{\mathrm {c}}|/B_{\mathrm {c}}$, $|d-d_{\mathrm {c}}|/d_{\mathrm {c}}$, $|\gamma -\gamma _{\mathrm {c}}|/\gamma _{\mathrm {c}}$, and $|\sigma -\sigma _{\mathrm {c}}|/\sigma _{\mathrm {c}}$, for the horizontal axis represent the normalized distance from the critical point, where $\gamma$ indicates the strain amplitude for colloidal suspensions and soft glasses, and $\sigma$ represents the stress for dislocations. Closed and open symbols denote the data in the reversible and irreversible states, respectively. Colored solid lines show the power-law fits of $\tau$ with $\nu$ indicated in the figure. Black solid and dashed lines represent slopes of $\nu =$ 1.295 and 1.110, respectively, which are predicted by the DP theory in 2D and 3D. As mentioned above, our data for the density-driven and shear-driven RIT fall on the red and blue solid lines, respectively, which give nearly the same slopes of $\nu = 1.32 \pm 0.10$ and $\nu = 1.38 \pm 0.08$^10^. They are, within error bars, in agreement with $\nu = 1.295 \pm 0.006$ predicted by the 2D DP theory^1,2^, consistent with the fact that our vortex system is a 2D system. On the other hand, in the colloidal suspensions and dislocations, $\nu = 1.1 \pm 0.3$^5^ and $\nu = 1.1$^43^ have been reported, respectively, which are smaller than $\nu = 1.295$ predicted for the 2D DP theory, but close to $\nu = 1.110$ for the 3D DP theory^1,2^. This result is reasonable, because the colloidal suspensions and dislocations are 3D experimental systems. Meanwhile, simulation of cyclically driven dislocations in the 2D system has found the exponent $\nu = 1.375$^11^, consistent with 2D DP, where the dislocations always organize into a reversible state both above and below the transition. The soft glass seems to be an exception since $\nu$ is $1.1 \pm 0.3$^42^ despite the 2D system. The authors of Ref.^42^ argue that RIT observed in the soft glasses may belong to another universality class known as the conserved DP class^2,44,45^, which predicts $\nu = 1.225 \pm 0.029$ in 2D.

It is also seen from Fig. 4 that for all the systems the relative width of the critical region spans the broad range up to at least 0.3, which is much larger than that of typical equilibrium critical phenomena^46,47^. The independence of the critical exponents on the driving parameters, *B* and *d*, observed in this work is somewhat surprising considering that the critical region is so large, which is clearly beyond the linear approximation.

Let us next focus on the data of $\tau (d)$ on the reversible side of the shear-driven RIT in our vortex system, which are plotted with closed blue squares in Fig. 4. We have noticed in Ref.^10^ that, when *d* is decreased down to around 25 nm($\equiv d_{\mathrm {c}1}$), which is close to the intervortex spacing $a_0 \approx 26$ nm at 3.5 T, in the reversible regime, $\tau (d)$ shows a downward deviation from the power-law relation $\tau \propto (d_{\mathrm {c}}-d)^{-\nu }$ with $d_{\mathrm {c}} = 45.2$ nm. This is marked by a dotted line in the main panel of Fig. 4. Here, we perform a detailed analysis of the data in the reversible regime, including additional data taken for $d<d_{\mathrm {c}1}$, which are shown in the inset of Fig. 4 as a locally enlarged graph. First, note the trivial fact that the upper limit of $|d - d_{\mathrm {c}}|/d_{\mathrm {c}}$ in the reversible phase is 1, which is given when $d = 0$. We find that, after showing the sharp, significant downward deviation at $d_{\mathrm {c}1}$, $\tau (d)$ for $d<d_{\mathrm {c}1}$ again follows the power-law function (the lowest blue line) that diverges at the common critical point $d_{\mathrm {c}}$ with a critical exponent $\nu = 1.30 \pm 0.18$, which are, within error bars, in agreement with $\nu = 1.38 \pm 0.08$ obtained for $d_{\mathrm {c}1}<d<d_{\mathrm {c}}$ and $\nu = 1.295 \pm 0.006$ expected for the 2D DP class. This result indicates that there are two types of the reversible states for $d>d_{\mathrm {c}1}$ and $d<d_{\mathrm {c}1}$, and that their critical behaviors with the same critical point $d_{\mathrm {c}}$ and exponent $\nu$ are identical to each other.

Recent molecular dynamics simulations predict two distinct reversible regimes referred to as point-reversible and loop-reversible states^16–18^. In the point-reversible state, particles self-organize into the arrangement in which they do not collide with each other. On the other hand, particles in the loop-reversible state experience multiple collisions even in the steady state but show the reversible behavior where non-affine loop trajectories are formed. The loop-reversible state has been proposed to lie between the point-reversible and the irreversible states. The loop-reversible behavior was not discussed by the original random organization model^5^, but later verified in a cyclically-sheared 2D soft-jammed material^48^.

We consider that these two reversible states are realized in our vortex system. The threshold particle numbers $N_{\mathrm {c}}$ at the shear-driven and the density-driven RIT, as defined as $N_{\mathrm {c}} \equiv d_{\mathrm {c}}/a_0$ and $N_{\mathrm {c}} \equiv d/a_{\mathrm {0c}}$, are 45 $\mathrm {nm}$/26 $\mathrm {nm} =$ 1.7 and 60 $\mathrm {nm}$/27 $\mathrm {nm}$ = 2.2, respectively, which are around 2. This means that RIT in our vortex system occurs when the vortices move about twice the intervortex distance. In the reversible regime near the RIT ($d_{\mathrm {c}1}<d<d_{\mathrm {c}}$), therefore, the vortex-vortex collisions survive even in the steady state, indicative of the loop-reversible state. On the other hand, for *d* below $d_{\mathrm {c}1}(\approx a_0)$, the collisions between the vortices are less frequent, so that the random-organization process is less effective, resulting in the significant reduction of $\tau$. Thus, this region ($d<d_{\mathrm {c}1}$) in the steady state corresponds to the point-reversible state. The similar drop in $\tau$ at the low strain amplitude has been found by computer simulation^18^. Figure S11 of Ref.^18^ indicates the transition or the crossover from the loop-reversible to point-reversible states, which occurs with a decrease in the strain amplitude.

As depicted with closed red circles in Fig. 4, in the density-driven experiment, we do not observe the downward deviation of $\tau (B)$ at low *B* that shows the point-reversible state. This is simply because the field *B* we can use in this experiment is limited to the regime of the peak effect, $B = 2-4.2$ T, where pinning is effective. Specifically, the shear amplitude $d = 60$ nm used here is always larger than $a_0 = 24{-}35$ nm, corresponding to $B = 4.2-2$ T. In order to study the point-reversible regime, we need low enough $B\,(< 0.65$ T) that satisfies the condition of $a_0(B) > d\,( = 60$ nm). However, it is difficult to measure the clear relaxation signal of |*V*(*t*)| in the low-*B* regime where pinning is not effective. Alternatively, when we used *d* smaller than 60 nm, we could not study the irreversible regime due to the lower limit of $a_{0}\,(\ge$ 24 nm), i.e., $B \le 4.2$ T, in this work.

We expect that both the point-reversible and loop-reversible states, and the transition or the crossover between them observed in the vortex system will be also observed in other many-particle systems. It has been observed in a dilute particle system of colloidal suspensions^4,5^ that the collisionless point-reversible state covers a wide range of the reversible regime. In the dilute system the loop-reversible state may possibly exist in the vicinity of the critical point. On the other hand, it has been found in a relatively dense particle system of soft glasses^42^ that the most part of the reversible regime corresponds to the loop-reversible state and the point-reversible state is expected to appear far away from RIT. The loop-reversible state in soft glasses contains many local plastic events, and their spatial correlations have been found to grow on approaching the yielding transition (or RIT)^42,49,50^.

To summarize, we study the critical behavior of RIT driven by the vortex density *B*, as well as the shear amplitude *d*, in the 2D vortex system. The fraction of active (colliding) vortices estimated from $V^{\infty } - |V(t)|$ exhibits a power-law time dependence with an exponent of *a* at the critical points of both types of RIT, $B_{\mathrm {c}}$ and $d_{\mathrm {c}}$. The relaxation times, $\tau (B)$ and $\tau (d)$, for the system to reach either the reversible or irreversible state show a power-law divergence at $B_{\mathrm {c}}$ and $d_{\mathrm {c}}$, respectively, with an exponent of $\nu$. We find that $a = 0.45 \pm 0.05$ and $\nu = 1.32 \pm 0.10$ obtained for the density-driven RIT are, within error bars, in agreement with $a = 0.40 \pm 0.05$ and $\nu = 1.38 \pm 0.08$ obtained for the shear-driven RIT^10^. The results show that independent of the driving parameters, *B* and *d*, both types of RIT fall into the same universality class as the absorbing transition in 2D DP, which predicts $a \approx 0.45$ and $\nu = 1.295 \pm 0.006$^1,2^. This is somewhat surprising considering that the critical region is so large, clearly going beyond the linear approximation.

As *d* is decreased in the reversible regime ($d < d_{\mathrm {c}}$) for the shear-driven RIT, $\tau (d)$ shows a sharp, significant drop at $d_{\mathrm {c}1}\approx a_{0}$, reflecting the suppression of vortex-vortex collisions and of random organization. This result indicates the transition or the crossover from the loop-reversible state with vortex-vortex collisions in the regime $d_{\mathrm {c}1}<d<d_{\mathrm {c}}$ to the collisionless point-reversible state in the regime $d < d_{\mathrm {c}1}$, as proposed by recent numerical simulations^16–18^. In either regime, $\tau (d)$ exhibits a power-law divergence at the same $d_{\mathrm {c}}$ with nearly the same exponent: $\nu =$ 1.38 and 1.30 for the loop-reversible and point-reversible states, respectively.

We expect that this work will stimulate further research on other absorbing phase transitions^51–55^ and phenomena related to RIT in various many-particle systems, including dilute colloids^5,25,56^, emulsions^26,27^, soft glasses^42^, jammed materials^57,58^, amorphous solids^8,59^, and skyrmions^20^. It is also of interest to study whether the critical behavior of the nonequilibrium transitions is independent of the driving parameters, specifically, the same critical behavior is observed in different nonequilibrium transitions, such as the depinning transition^3,39,60–63^ and the clogging transition^64^, with the driving parameter being the particle density.

## Methods

### Sample and experimental setup

The strip-shaped film of *a*-$\hbox {Mo}_{x}\hbox {Ge}_{1-x}$ ($x \approx 0.77$) with the thickness of 330 nm was fabricated by radio-frequency sputtering deposition onto a Si substrate mounted on a water cooled copper stage that rotated at 240 rpm^10^. The superconducting transition temperature $T_{\mathrm {c}}$ at which the resistivity falls to zero was 6.3 K at $B = 0$ T. The vortices were induced by applying *B* perpendicular to the film surface. The size of the vortex core and the range of repulsive vortex-vortex interaction characterized by the superconducting coherence length and London penetration depth are estimated to be $\approx 1 \times 10$ and $\approx 10^{2}$ nm, respectively^65^. By applying the current, the vortices move in the direction parallel to the film width of 0.3 mm. The voltage *V* induced by vortex motion was measured by a standard four-probe method using voltage probes separated at $l=$ 1.2 mm. The film was directly immersed into the liquid $^{4}$He and all the experiments were conducted at 4.1 K. The vortex density $(1/a_{0})^{2}(=\sqrt{3}B/ 2\Phi _0)$ was varied from $(1/35)^{2}\,\hbox {nm}^{-2}$ to $(1/24)^{2}\,\hbox {nm}^{-2}$ by changing *B* from 2.0 to 4.2 T, where $a_0 = (2\Phi _0/\sqrt{3}B)^{1/2}$ is the average intervortex spacing.

### Measurements

The initial vortex configuration was prepared by shearing the vortices for a long time, typically more than 4000 cycles, with shear amplitude of $d = 3\,\upmu \hbox {m}$ sufficiently larger than the critical shear amplitude $d_{\mathrm{c}} \approx 45$ nm obtained in Ref.^10^. This protocol randomizes the configuration of vortices due to the diffusivity in the irreversible state. The frequency *f* of the ac square current $I_{ac}$ for initialization was 4 kHz and the amplitude of $I_{ac}$ was adjusted to generate the desired value of the steady-state voltage $V^{\infty } (\equiv |V(t \rightarrow \infty )|)$ that yields $d = V^\infty /2flB = 3\,\upmu \hbox {m}$ for each *B* studied here. The time-evolution of the voltage *V*(*t*) immediately after applying $I_{ac}$ to the initial configuration was measured using an oscilloscope (Rohde and Schwarz RTO2024) with resolution of 10 MHz. The amplitude of $I_{ac}$ for random organization was adjusted to generate $V^{\infty }$ satisfying $d = 60$ nm, while *f* was fixed at 200 kHz. According to the relation $d = v/2f$, this condition of constant *d* with fixed *f* gives the same average velocity *v* of vortices for different *B*, which allows us to neglect the influence of the velocity on RIT^66^.

## Data Availability

The data that support the findings of this study are available from the corresponding author upon a reasonable request.
